# Supplementary material for: On the identification of differentially-active transcription factors from ATAC-seq data
Source: PLoS Comput Biol. 2024 Oct 23;20(10):e1011971. doi: 10.1371/journal.pcbi.1011971 (PMC11534267; doi:10.1371/journal.pcbi.1011971)
Supplement: Attachment — Submitted filename: DTFAB responses.docx [file pcbi.1011971.s003.docx]

# Point-by-point response to reviewers

We thank the reviewers for their valuable feedback. Several new analyses were requested, and we’ve strived to satisfy all of them. Below are the original comments from each reviewer in *black*, followed by our *answer in blue* below each point.

# Reviewer #1:

The study of Gerbaldo et al. describes a thorough benchmarking study comparing methods for inferring the activity of transcription factors from differential chromatin accessibility between conditions, such as protein knockout or overexpression.

 The overall design and structure of the study are very careful, and the execution of the analysis seems very convincing, from exhaustive supplementary figures to the organization of the underlying data and code. I strongly support the publication of the manuscript, as, despite the narrow topic and heavily technical nature of the study, this is a great practical example of performing a comprehensive benchmarking study, which includes simulations, previously published data, and a newly generated experimental dataset.
 I consider the manuscript to be mostly publication-ready, although introducing minor edits could be useful here and there. Please find more details below. Of note, I am not a native English speaker thus some of my suggestions might be non-relevant.

 Sincerely yours,
 Ivan V. Kulakovskiy

 General
Throughout the text, you use the term 'motif' ambiguously: (1) to denote the model of the DNA pattern describing the TF-DNA binding specificity and (2) to denote particular pattern occurrences in the sequences of ATAC-Seq peaks. I strongly suggest differentiating the respective objects explicitly: please use the term 'motif' or 'motif model' to describe the pattern (e.g. the position weight matrix) and use 'motif hits' or 'motif occurrences' or 'motif matches' when talking about particular 'words' yielded by sequence scanning (motif finding, pattern matching). This ambiuity is a general issue in the literature and would be great to avoid. Particularly, this makes it difficult to properly understand the description of the 'network score' (line 237).

>> We thank the reviewer for this comment. We’ve tried to clarify the usage throughout the paper, most of the time specifying either “motif match” or “motif model”. In some places however it was impractical to do so, but we have at least made sure that any instance where motif matches are specifically meant were clearly indicated as such, while we took ‘motif’ on its own as by default meaning the general binding pattern, rather than its specific occurrences.

Another general problem comes with 'data not shown' statements, e.g., it is unclear what you mean by 'the best results' (line 127) or what are the 'other technical sources of variation' (lines 413-414). Please consider briefly describing the underlying data e.g. with a few examples.

>> We now clarified this.

 Suggested additions
 While the technical results are described in a very detailed way, the Discussion could include a special section dedicated to interpreting the observed effects. First, regarding the NFKB1 TRAFTAC data: should we treat the low-to-inconsistent effect for the NFKB1 motif as a typical outcome given what is known on its cellular role? Finding the members of the AP-1 complex among the most significant TFs linked with the differential chromatin accessibility is not surprising, and a brief comparison to the other benchmarked datasets could be informative, i.e., whether those motifs are commonly detected on top of the list. Exploring the composition of the typical 'false positives' could be also informative in this context.

>> We thank the reviewer for this interesting idea. To investigate whether some motifs other than the perturbed TF tended to be recurrently found across perturbations in the benchmark datasets, we first used the intersection of those called as significant by the top variant from both chromVAR and monaLisa. Only a single motif, ELF5, occurred in more than one dataset (in BANP and GATA1). Reasoning that this might be too stringent, we took the intersection of the top 20 motifs from each of the two methods (irrespective of their significance). Again, no motif occurred in more than two datasets (those that did are ELF5, GATA4, GATA6, and SMRC1).

We finally directly checked the occurrence of JUN/FOS family members. With the exception of the BANP dataset, no such motif was found in the top 30 motifs by both methods, and only in 3 datasets (BANP, GATA2, and NR1H4) by one of the two methods. Therefore, despite the key role of the AP-1 complex in both enhancer activation and dynamic response to stimulus, we did not find evidence for its widespread disruption across the benchmark perturbations, and the effect appears to be genuinely linked to the TNFa-NFKB1 pathways. We now note this in the discussion of these results.

Similarly, it seems interesting to hear your interpretation of striking differences between close family members, such as GATA1-GATA2 and RUNX1-RUNX2. Is it a direct consequence of a limited overall magnitude of change in chromatin accessibility for one of two similar TFs? Could it be linked to a difference in protein expression? This might be helpful to highlight and explain the 'difficult' datasets, especially for pairs of TFs sharing similar binding specificities and, thus, similarly affected by any issues related to motif scanning.

>> As we discussed, this is to some extent reflected by the extent of peak-level changes: the GATA1 dataset for example shows massive differences in accessibility, while nothing reaches genome-wide significance in the GATA2 dataset, on the other hand neither RUNX dataset shows peak-level changes passing FDR (see Supplementary Figure 1A.). In the RUNX1/2 case, this could be explained by expression: while RUNX1 is highly-expressed at the RNA level in this system, RUNX2 is lowly expressed – if it was not very active in the first place, its knockdown is unlikely to have a big effect on accessibility. We now mention this in the manuscript. In the GATA1/2 case however, both are very highly-expressed at the level of RNA (we did not have at hand a proteomic dataset detecting them). Finally, as these are all datasets based on genetic deletion, a critical factor is likely to be the extent to which the protein is depleted after 72h, which in turn depends on the stability of the protein and RNA. Given that, beyond the specific case of RUNX2, we do not have any solid explanation, we did not venture further on the question.

 Motif scanning
 Using FIMO with default settings is appropriate as an off-the-shelf approach, but exploring if using strict or weaker thresholds would affect the 1-2 top-scoring methods might be informative, e.g., using NFKB TRAFTAC data as a brief case study. This might be particularly interesting given monaLisa's solid performance and internal motif-finding procedure.

>> We thank the reviewer for this suggestion. First, we controlled that the global number of motif matches identified by monaLisa’s internal matching procedure was similar to those called for other methods. Indeed, across the benchmark datasets, the number of motif matches used by monaLisa was within a 10-20% difference to the number of fimo matches used for other methods. We now note this in the relevant section of the methods.

Following the reviewer’s suggestion, we ran a selection of the top methods across the benchmark datasets using a more stringent motif matching threshold. The results (shown in Supplementary Figure 6) indicate that the methods are relatively robust to such change, although the more stringent matching tended to have a slightly lower power.

 Minor comments regarding the text
 The following word choices seemed strange to me and I suggest rechecking and, if necessary, rephrasing the respective statements and/or providing more details:
 'analytic variations' // Author summary -> changed
 'the impacts of a novel method' // Author summary -> here we’re not quite sure what the problem is, we simply changed for the ‘effects of’ the TRAFTAC.
 'regulate a given transcriptional signature' // line 4 -> same here, not sure what the problem is
sonja 'less biased' // line 21 -> same
 'peak samples' // line 68 -> fixed
 'each rule' // lines 146-147 -> this formulation is from the diffTF documentation, but anyway as this is the default setting we now left it out.
 'we had some difficulties running the code' // lines 197-198, please provide some details -> we now clarified this.
 'Benjamini, Hochberg, and Yekutieli' // lines 247-248, do you mean "BH" (aka "FDR" ) or "BY"? -> thank you for flagging this mistake, it is indeed BH.

 Minor comments regarding Figures and Figure labels
 Figure 1: Please provide an extra (supplementary?) table summarizing all variants of methods assessed in the benchmark (rows in panel A). The current naming scheme is not uniform and hard to track throughout the manuscript. FDR on panel B (Y-axis) can be also mistaken for adjusted P-values. Please also spell out the plot labels, i.e. "Adj. P-value" instead of "p.adjust". -> we made the requested changes.
 Figure 2: please rephrase 'true motif ranks' to reduce ambiguity. It can be read as 'ranks of true motifs' or 'true ranks of various motifs', which have dramatically different meanings in the context of your benchmarking setup. -> we’ve now tried to clarify this throughout the paper
 Figure 5: 'simes' should be probably capitalized. -> fixed, thanks

# Reviewer #2

The manuscript titled "On the Identification of Differentially Active Transcription Factors from ATAC-seq Data" delves into the performance evaluation of TF enrichment analysis methods. The authors aimed to determine the optimal pipeline for TF enrichment by benchmarking background selection, variability normalization, incorporation of factors in multivariate linear models for differential analysis, and the combination of multiple tools to adjust p-values. It's noteworthy that quantile normalization yielded the best results for z-score normalization.

 TF analysis presents a challenging question to address within a single manuscript, and it's not surprising that this study leaves several questions unanswered. Key steps in TF enrichment analysis include motif selection, definition of binding sites, fragment count, background correction, counts normalization, model selection, and enrichment analysis. While the authors covered most of these steps, there's a lack of thorough discussion on motifs. This oversight led to some expected results, as noted in lines 587-595. The authors should explain why they chose HOCOMOCO over other databases and address how motifs generated from different methods could introduce bias in TF binding site prediction. Additionally, merging similar motifs is a crucial step in motif cleaning that should not be overlooked.

>> This is a valid concern, and as we had mentioned in the discussion, the motif collection used is a critical factor which we left out, for the simple reason that we can’t investigate everything at once. It is very probable that a better motif collection would lead to better results in general, however our reasoning was that the relative performance of the methods compared to each other should be relatively stable to changes in the motif collection used (although we now discuss more clearly the biases this can introduce). Following this logic, while HOCOMOCO is not the only reasonable choice, it seems an unproblematic one: while it is certainly imperfect, it is one of the most widely-used and largest collection of motifs with reduced per-TF redundancy; in addition it is based on a standardized pipeline (as opposed to collections of motifs derived through very heterogeneous means) and integration based on multiple types of experimental data (e.g. as opposed for instance to SELEX-only, which might be biased relative to in vivo binding patterns).

While it is not the aim of our present study to evaluate different motif collections, we tried to address the reviewers’ comments by expanding our analyses to consider motif similarity. Briefly, we clustered similar motifs into archetypes, computed another evaluation metric based on these (shown in Figure 1, and which largely agrees with the ranks), computed precision considering similarity, and finally repeated the benchmark using the archetypes directly, for a selection of the methods (Supplementary Figure 7).

 It's important to recognize that there is no ground truth for TF enrichment analysis. The authors attempted to create a method for generating "true TFs," which could be a significant advancement in the field. Further discussion on "true TFs" in the manuscript, including aspects such as the distance distribution relative to transcription start sites and co-localizations among the binding sites of "true TFs," would greatly enhance the depth of the analysis.
>> We now further discussed the issue of the ‘true TF’ in the manuscript. Further than that, we are afrad we did not quite understand what the reviewer was asking for, or how it would affect the main results presented. (We did investigate motif colocalization in the simulations in relation to detection difficulty).

Would it be beneficial to consolidate the software used in the comparison into a summarized table for clarity?

>> We now added a supplementary table describing all the method variants.

 Additionally, it's intriguing to note that all footprint-based methods did not yield results for the "true TF." I wonder if the footprint-based method could potentially compensate for biases in chromatin accessibility-based methods. Could it be feasible to include the footprint for the NFkB motif under both conditions in the section discussing TRAFTAC-mediated TF degradation?

>> We are not sure we understand the reviewer here: certainly, some of the footprint-based methods (e.g. insertionModel) performed relatively well. Multiple studies have now shown that the majority of TFs do not leave a clear footprint in ATACseq data. In light of this, it is plausible that they would be highly efficient for some factors (e.g. pioneers, CTCF, etc.), while not well adapted to the majority. Indeed, the insertion profile around the NFKB1 motifs in the TRAFTAC dataset (below) for instance looks anything like a footprint. It shows a mild (if highly consistent across replicates) reduction, but in the absence of footprint (even upon Tn5 bias correction) there cannot possibly be a change in its relative depth etc.

We wish to make clear that the characterization of the TRAFTAC experiment is offered as an illustration of the methods in a largely explorative context. To our knowledge, this is the first time accessibility is profiled following such a treatment, and although there is a clear downregulation of the protein, much remains to be explored as to the specificity and functional consequences of TRAFTAC-mediated interventions.

Here are some minor points that need to be addressed:

 1. Detailed parameters are missing in lines 62-72. >> We added the exact parameters.
 2. I couldn't find a description for reads shift in the method, although the author may have mentioned it. >> The bam files themselves were not shifted. Read shifting is of relevance to methods that use the insertion sites positions (i.e. ATACseqTFEA, insertionModel, and BagFootLike), all of which shift reads internally by default.
 3. If possible, could you add the TFEA (https://doi.org/10.1038/s42003-021-02153-7) package to the comparison? >> We had tried to run it before submission, and were unable to. We now tried again on two separate servers, using inputs at different steps from different datasets. We tried to debug, but were still unable to get results: it systematically crashes due to a memory allocation problem, either after a few hours or after running for over 5 days. One of the errors has already been filed on github some years ago, with no solution (issue 17).

We note however that the study from Santana et al. (a related, though complementary study using especially H3K27ac, published during the revision process, and which we now compare to in the manuscript) included TFEA, and it did not have particularly strong performance. So instead we added another method, MEIRLOP, which was among the top methods in that study.
 4. Would it be better to describe fold-changes by formula in line 268? >> We tried to clarify the according paragraph and added a formula describing how the fold changes were introduced in the semi-simulations.
 5. Many figure legends are missing, such as Figure S2D and Figure S4. >> We are not sure what the reviewer is missing here; certainly we did not see any missing legend in Figure S4. In Figure S2D, there is no legend for what colour corresponds to each method, but given the large number of methods this would take a lot of space and is irrelevant to make a point about the metrics, where we only wanted to show that disagreements are dataset- rather than method-related (the specific values for the specific methods can be obtained for instance from Figure S6).
 6. How did the authors conclude that the two metrics show strong agreement in Figure S2D? >> We now clarified this in the figure legend. We also fixed a bug which resulted in an over-optimistic AUC for some methods that fail to report a score for some TFs, and updated Figure 1 accordingly, resulting in an even clearer agreement between metrics.
 7. There are some typos, such as "FigS2B" in line 371 and "FigS3C-E" in line 426. >> We are sorry but we fail to see any typo there.
 8. There seems to be a figure repeated in Fig4AB and FigS7B. >> We thought that this would help the reader, given that the latter is largely a more detailed version of the former. We now mention this explicitly in the figure caption to avoid any confusion.
 9. It's challenging to see the red circles for NFKB1 and NFKB2 in Fig5A. >> We changed the colour to increase visibility.

# Reviewer #3

The authors present a very nice benchmarking study for testing computational tools for identifying TF motif activity based on ATAC-seq data. They carefully selected a set of studies that specifically perturb only one TF ano d then measured ATAC-seq data. To further validate their findings, they simulated the removal of a TF by using ChIP-seq data of the TF to predict where it binds, and then specifically down sampled the ATACseq signal at these loci. They have done a great job in assembling a very thoughtful ground truth, both simulated and real data.

 Major comments:
 - while the authors tested the whole range of methods on the real data, they only tested a couple of methods on their simulated data. The simulated data, however is much better controlled in terms of what effects are expected, so it would be important to show all methods on the simulated data. One caveat of the real data - especially the K/O datasets that had the TF down for 72h is that there may be lots of indirect effects as well so testing the methods on the simulated data would be more fair to compare all method, because there, the authors know exactly what to expect.

>> Given the large number of simulated datasets and methods (some of which are very long to run), this took several weeks to compute, but we’ve now ran all methods on the semi-simulations as well, with the exception of ATACseqTFEA, which was very long to run, performed poorly on real datasets, and failed to run on some. This largely supported the results from the real datasets. Further, we were not able to run diffTF on two of the 32 semi-simulated datasets, we reported the error as an issue on the bitbucket repository of diffTF (<https://bitbucket.org/chrarnold/difftf/issues/81/error-in-rule-intersectpeaksandbam>). As we obtained results from diffTF for almost all of the simulated datasets, we were still able to get a thorough impression of its performance.

We updated main Figures 4 and 6 with further methods and added supplementary Figure S15 showing the performance of all methods except ATACseqTFEA on the semi-simulated data with varying perturbation strength.

- The authors mention they ranked the TFs by fold-change, did they consider absolute fold-change? This is particularly important for TFs that may act as repressor in the real datasets (not in the simulated) because one would expect a loss of accessibility for repressors (as shown e.g. in the diffTF paper). Maybe the reason Runx2 is not detected by any method is because it is a repressor and accessibility is lost upon its deletion?

>> Yes, we did rank by absolute logFC or coefficient (after significance), as is indicated in the methods.

 - diffTF should not be used in permutation mode for these small sample sizes, this is completely meaningless. Also it is meaningless to increase permutations to 1000, that just means many permutations will be exactly the same

>> As a matter of fact it did not perform much worse than the analytic mode, indicating that it was not meaningless to test. Nevertheless, we now removed it from the main figure, and only kept it in the supplementary figure where all variants are shown. We also further discuss this in the methods section, and mention in the discussions that diffTF(permutations) could show a better performance in larger datasets.

 - The authors should emphasise better that ChromVar only performs well when combined with their additional extension using the limma model, it should be very clear that ChromVar on its own (I’m assuming that’s chromVAR:differentialDeviations) performs quite badly. This is important for readers to understand, not that they use chromVar in default.

>> We are sorry that this did not come out as clearly as we intended. We now tried to emphasise this further.

 - Many TFs have similar motifs, the authors should consider this, especially for the precision measurement in Fig 1B, but maybe also for the rank based analysis. I could envision a similar analysis as the network based analysis (which is very cool by the way!) could be done for TFs with similar motifs

>> This is indeed a very good suggestion and we did exactly that. First we clustered similar motifs into archetype (see methods), then computed an archetype score which, similar to the network score, computes a relative AUC of the CAT plot using motifs that belong to the same archetype, instead of cofactors. This is now presented in Figure 1A along with the other scores (with which it is largely in agreement). In addition, as suggested we computed precision in Figure 1B by counting as a positive any motif that belongs to the archetypes of any network member, i.e. true TF plus interactors (these were however not used for sensitivity estimates). The impact on the precision estimates is extremely small, which we take as a positive result, and we nevertheless believe that this change makes the precision more interpretable.


 Minor comments:
 - the references are not sorted according to appearance

>> They are now in order of appearance.

- the ordering in Figure 1A is not very clear, how were the methods ordered? It seems some of the well-performing methods are lower than some of the more poorly performing methods. If it is based on the sum of the ranks, maybe the Runx2 should be taken out to avoid skewing based on arbitrarily high ranks?

>> In order to reduce the impact of high ranks, the methods were sorted based on the mean of the sqrt of the ranks (i.e. not using the network score). We now changed this in two ways: first we rank methods based on all three displayed metrics; second, to combine the ranks with the other metrics (which are on a completely different scale), we use a transformation based on the inverse logit of the sqrt of the ranks (which nicely decays towards zero for higher ranks):

( where x is -sqrt(rank) )

- I’m wondering whether it is necessary to have all the variations of one method in the main figure, or whether it would be sufficient to show the default and the best-performing addition to one method in the main figure and the rest in the supplement. It would make the main figure 1 easier to read - but this is of course a decision the authors should take.

>> We agree; we now included only the original & top variant method in the main Figure 1 (and all variants in the supplementary figures).
